# Variation of endosymbiont and *citrus tristeza virus* (CTV) titers in the Huanglongbing insect vector, *Diaphorina citri,* on CTV-infected plants

**DOI:** 10.3389/fmicb.2023.1236731

**Published:** 2023-09-22

**Authors:** Xiaoqing Cui, Yangyang Liu, Jingtian Zhang, Panpan Hu, Zheng Zheng, Xiaoling Deng, Meirong Xu

**Affiliations:** Guangdong Province Key Laboratory of Microbial Signals and Disease Control, South China Agricultural University, Guangzhou, China

**Keywords:** Huanglongbing, Asian citrus psyllid, citrus tristeza disease, acquisition, persistence, *Wolbachia*, host preference

## Abstract

“*Candidatus* Liberibacter asiaticus” (CLas) is a notorious agent that causes Citrus Huanglongbing (HLB), which is transmitted by *Diaphorina citri* (*D. citri*). We recently found that the acquisition and transmission of CLas by *D. citri* was facilitated by *Citrus tristeza virus* (CTV), a widely distributed virus in the field. In this study, we further studied whether different CTV strains manipulate the host preference of *D. citri*, and whether endosymbionts variation is related to CTV strains in *D. citri*. The results showed that the non-viruliferous *D. citri* preferred to select the shoots infected with CTV, without strain differences was observed in the selection. However, the viruliferous *D. citri* prefered to select the mixed strain that is similar to the field’s. Furthermore, *D. citri* effectively acquired the CTV within 2–12 h depending on the strains of the virus. The persistence period of CTV in *D. citri* was longer than 24 days, without reduction of the CTV titers being observed. These results provide a foundation for understanding the transmission mode of *D. citri* on CTV. During the process of CTV acquisition and persistence, the titers of main endosymbionts in *D. citri* showed similar variation trend, but their relative titers were different at different time points. The titers of the “*Candidatus* Profftella armatura” and CTV tended to be positively correlated, and the titers of *Wolbachia* and “*Candidatus* Carsonella ruddii” were mostly negatively related with titers of CT31. These results showed the relationship among *D. citri*, endosymbionts, and CTV and provided useful information for further research on the interactions between *D. citri* and CLas, which may benefit the development of approaches for the prevention of CLas transmission and control of citrus HLB.

## Introduction

Citrus tristeza disease is one of the most common and economically important citrus virus diseases in citrus around the world, which is caused by *citrus tristeza virus* (CTV). It is widely distributed in more than 50 countries across the Asia-Pacific, Europe, Americas, Africa and the Mediterranean (Moreno et al., 2008; Ghosh et al., 2022). The hosts of CTV are mainly *Citrus* and *citrus* relatives in the family Rutaceae, such as *C. auantium*, *C. sinensis*, *C. limon* and other varieties (Roistacher and Bar-Joseph, 1987). Different varieties of citrus plants have different degrees of susceptibility to CTV and show different intensities of symptoms when infected. CTV is highly diverse in genome. According to the classification method of CTV strains by Hilf et al. (2005), Zhou et al. (2008) confirmed the CTV strain CT31 has 3 genotypes simultaneously: T30, VT and T3. CT31 is a mild strain contains two haplotypes: CT31-1 and CT31-2 (Wang et al., 2017). However, YLH is a mixture of different CTV genotypes, which is similar with the CTV in the field. The symptoms caused by different strains of the virus are different. CTV-susceptible hosts including sweet orange (*Citrus sinensis*) propagated onto sour orange (*C. aurantium* L.) rootstocks, grapefruit (*C. paradisi* Macf.), Mexican lime (*C. aurantiifolia*) or *C. macrophylla* Wester (Roistacher and Bar-Joseph, 1987; Gómez-Muñoz et al., 2016), which causes the severe quick decline (QD), seedling yellow (SY) or the surface of stem xylem to produce stem pitting (SP) (Rocha-Peña et al., 1995; Suastika et al., 2001). CTV-tolerant hosts include most lemons (*C. limon*), the mandarins (*C. reticulata*), etc. *Poncirus trifoliata* is one of the CTV-resistant hosts (Bar-Joseph et al., 1989).

CTV is mainly transmitted by seedling transportation, vector feeding and scion grafting (Bar-Joseph et al., 1977; Wu et al., 2021). So far, it has been believed that the vector of CTV in the field is various aphids (Bar-Joseph et al., 1977). These aphids included *Aphis* (*Toxoptera*) *citricidus* (Kirkaldy), *Aphis gossypii* Glover, *Aphis spiraecola* Patch, and *Aphis* (*Toxoptera*) *aurantii* (Boyer de Fonscolombe) (Bar-Joseph et al., 1989), among which the *Aphis* (*Toxoptera*) *citricidus* was recognized as the most efficient vector. Bar-Joseph et al. (1989) proposed that *Aphis* (*Toxoptera*) *citricidus* could acquire CTV and have the ability to transmit virus after feeding on CTV-infected citrus for 30 min. *Aphis* (*Toxoptera*) *citricidus* can spread a variety of CTV strains, and its transmission efficiency of severe CTV strains is higher than that of mild CTV strains (Broadbent et al., 1996).

*Diaphorina citri* (*D. citri*) is the vector of *Citrus* Huanglongbing, the most severe bacterial disease in the world’s citrus industry mainly caused by “*Candidatus* Liberibacter asiaticus” (CLas). Both CLas and CTV are phloem-limited and are carried by *D. citri*. Britt et al. (2020) used high-throughput sequencing technology to detect the presence of a variety of viruses in the *D. citri* population in Florida, United States, among which CTV was widely present and highly abundant in the *D. citri* population in the field. In recent years, our laboratory has found that *D. citri* can acquire and carry CTV both in the field and in the laboratory conditions (Wu et al., 2021). Chen et al. (2023) speculated that CTV could break through the midgut barrier and replicate in the midgut of *D. citri*, which was different from the non-circulating semipersistent manner of aphids in transmitting CTV. Intriguingly, *D. citri* carrying CLas probably had no significant effect on its transmission of CTV, however, *D. citri* carrying CTV further facilitated its acquisition and transmission of CLas (Chen et al., 2023). Based on these, we speculate some interaction between *D. citri* and CTV that must mediate the CLas and *D. citri* interaction.

In the process of acquiring and transmitting pathogens, vector insects are attracted by the composition and odor of plants. As tools of information transmission between insects and plants, the volatile organic compounds (VOCs) produced by plants can directly affect the feeding, reproduction, courtship and other behaviors of insects (Turlings and Erb, 2018). Plant pathogens can also indirectly change the feeding tendency and feeding behavior of insects by changing the proportion of nutrients, resistance strength and concentration of secondary substances of the host (Mauck et al., 2016, 2018; Eigenbrode et al., 2018). For example, plants infected with *Cucumber mosaic virus* (CMV) increase in ethylene and fatty acid precursor volatiles, which eventually attract aphids to feed, facilitating the spread of CMV (Mauck et al., 2014; Collum and Culver, 2016). Similarly, the activity of plant defensive enzymes and callose decreased in plants infected with tomato yellow leaf curvelet virus (TYLCV) could increase the survival rate of *Bemisia tabaci* (Su et al., 2015).

Endosymbionts of insects actively participate in many aspects of host life cycles that influence the biological traits of their host insects (Baumann, 2005; Engelstädter and Hurst, 2009; Feldhaar, 2011). They also play an important role in the nutrition contribution and withstanding the colonization of the gut by non-indigenous species including pathogens of the respective vectors (Dillon and Dillon, 2004). A series of studies have shown that endosymbionts involved in mediating transmission of pathogens in host insect vectors (Morin et al., 1999; Banerjee et al., 2004; Kambris et al., 2009; Hancock et al., 2011). Endosymbionts of insects can assist in the entry of pathogens into the insect. For example, the GroEL protein produced by endosymbiont in *Bemisia tabaci* protects TYLCV from destruction during the passage of the virus through the hemolymph (Morin et al., 1999).

There are a variety of endosymbionts in *D. citri*, among which “*Candidatus* Carsonella ruddii,” “*Candidatus* Profftella armatura” and *Wolbachia* are highly abundant and ubiquitous in *D. citri* (Chu et al., 2016; Jiang et al., 2023). “*Ca.* Carsonella ruddii” provides essential amino acids to host insects (Thao et al., 2000; Nakabachi et al., 2006). “*Ca.* Profftella armatura” can produce polyketide toxin with defensive functions, and provide a certain defensive role for the host insects (Nakabachi et al., 2013). *Wolbachia* can regulate the host reproduction, provide nutrition and regulate the host response to pathogens or biotic stress (Stouthamer et al., 1999; Werren et al., 2008; Li et al., 2013; Hussain et al., 2017).

Endosymbionts are influenced by different biotic and abiotic factors. The complex bacterial communities presumably play an essential role in the fitness of their insect hosts. A previous study comparing adult *D. citri* fed on CLas-infected plants and CLas-free plants found that “*Ca.* Profftella armatura” and “*Ca.* Carsonella ruddii” titers were significantly reduced in CLas-exposed males compared with those of unexposed males, but the titers of these endosymbionts were increased in the ovaries of CLas-exposed females, and *Wolbachia* titers were highest in the Malpighian tubules (Hosseinzadeh et al., 2019). Studies have also shown that there was a positive correlation between *Wolbachia* titers and CLas titer (Fagen et al., 2012; Jiang et al., 2023), and *Wolbachia* titers were higher than CLas titer (Kolora et al., 2015). However, the effects of CTV on the endosymbionts of the psyllid have not been examined. Considering the potential contributions of endosymbionts to the fitness of the psyllid and to the transmission of CLas, they might be associated with CTV, which favors the acquisition and transmissiton of CLas in *D. citri* (Wu et al., 2021). Therefore, it is necessary to elucidate the relationship between main endosymbionts and CTV in *D. citri*.

In conclusion, *D. citri* is the transmission vector of CLas, and CTV in *D. citri* can promote the acquisition and transmission of CLas. The main endosymbionts, “*Ca.* Carsonella ruddii,” “*Ca.* Profftella armatura” and *Wolbachia*, also regulated the interaction between *D. citri* and CLas. Different strains of CTV may play different roles in *D. citri*-CLas interaction by influencing the behavior of *D. citri* or altering the endosymbiont abundance of *D. citri*. Consequently, this study determined the selection preference of *D. citri* for citrus with and without CTV, analyzed the relationship between three endosymbionts of *D. citri* and CTV, and compared the acquisition and persistence rates of two CTV strains by *D. citri*, intending to provide a reference for clues to explain why CTV affects the transmission of CLas by *D. citri.*

## Materials and methods

### Plants, insects, and their cultivation conditions

Two-year-old healthy orange jasmine (*Murraya paniculata* L.) plants, “Shatangju” mandarin (*Citrus reticulata* Blanco ‘Shatangju’) plants were incubated in a temperature-regulated greenhouse under a 14 h:10 h light–dark cycle at 25 ± 1°C and 65% ± 2% relative humidity (RH). Scions with CTV strains of CT31 and YLH, provided by Dr. Yan Zhou from Citrus Research Institute of Chinese Academy of Sciences, were grafted on healthy “Shatangju” mandarin rootstock seedlings by side grafting. After three months, the average Ct value of CT31-infected seedlings in CTV detection was 20.84 ± 0.72, and that for YLH-infected trees was 20.92 ± 0.54.

*D. citri* adults collected from the campus of SCAU were used to establish psyllid colonies on orange jasmine plants in a temperature-regulated greenhouse for more than 10 generations (Wu et al., 2021). Ten individual psyllids were screened for the absence of both CLas and CTV every month by qPCR. The stock *D. citri* individuals without detachable pathogens were further used for the experiments.

### DNA, RNA extraction, and cDNA synthesis

E.Z.N.A.® Tissue DNA Kit (Omega Bio-tek., Norcross, GA, United States) was used to extract DNA from insect individuals. Total plant RNA was extracted from citrus leaves using E.Z.N.A.® Plant RNA Kit (Omega Bio-tek., Norcross, GA, United States). Total insect RNA was extracted from a single *D. citri* using TRlzol® Reagent (Life Technologies, Guangzhou, China). The concentration and purity of total DNA or RNA samples were quantified by absorbance using NanoDrop™ One micro-volume UV–Vis spectrophotometer (Thermo Scientific, Shanghai, China). The total RNA samples were individually used for reverse transcription using Verso cDNA Synthesis (TransScript) Kit (TransGen Biotech, Beijing, China). cDNA samples were stored at −80°C for further use.

### Real-time quantitative PCR (qPCR)

In this study, SYBR Green method was used for qPCR. The primer sequences for CTV and for the three endosymbionts detection were shown in Table 1. qPCR was carried out in 20 μL reaction mixtures containing 10 μL of SYBR Green Mix (TransGen Biotech, Beijing, China), 8 μL of ddH_2_O, 0.5 μL of forward primer (10 μM), 0.5 μL of reverse primer (10 μM) and 1 μL of template DNA or cDNA (~50 ng). The qPCR cycling parameters were 95°C for 2 min, 40 cycles of denaturation at 95°C for 15 s, and 20 s extension at 60°C. All qPCR reactions were performed in triplicate. Samples with Ct values lower than 33 were judged as CTV positive. At the same time, in order to relatively quantify the CTV or endosymbiont titers, standard curves were drawn using pEASY-T1 (TransGenBiotech, Beijing, China) recombinant cloning plasmids containing the target fragments and the relative primers, with 8 gradients (10(7)-fold). CTV titers was assessed by the copy number of CTV in per ng cDNA using a formula referencing to the study of Ruiz-Ruiz et al. (2009). β-actin was selected as endogenous internal control. The relative microbial titers was calculated by the method of 2^-△△CT^ (Livak and Schmittgen, 2001).

**Table 1:** Details of primers used for qPCR assay in this study.

Target species	Primer name	Primer sequence(5′ → 3′)	References
*Citrus tristeza virus*	cquctv1	TATAGAGGCGAAACTGCGAAT	Liu et al. (2008)
	cquctv2	CCTCATAACGAAGAAGCCCA	
β-actin	β-actin F	CCCTGGACTTTGAACAGGAA	Tiwari et al. (2011)
	β-actin R	CTCGTGGATACCGCAAGATT	
*Candidatus* Carsonella ruddii	*Myc*-F	TGGGAACGCCATATGCTAAT	Dossi et al. (2014)
	*Myc*-R	GTCCCAATGGGTTGTTCATC	
*Candidatus* Profftella armatura	*Syn*-F	GCCTTTATGGGTAGGGCTTC	Dossi et al. (2014)
	*Syn*-R	CCGGACTACGATGCACTTTT	
*Wolbachia*	*FtsZ*-F	AGCAGCCAGAGAAGCAAGAG	Dossi et al. (2014)
	*FtsZ*-R	TACGTCGCACACCTTCAAAA	

### Plant selection preference assay

**Figure 1:**
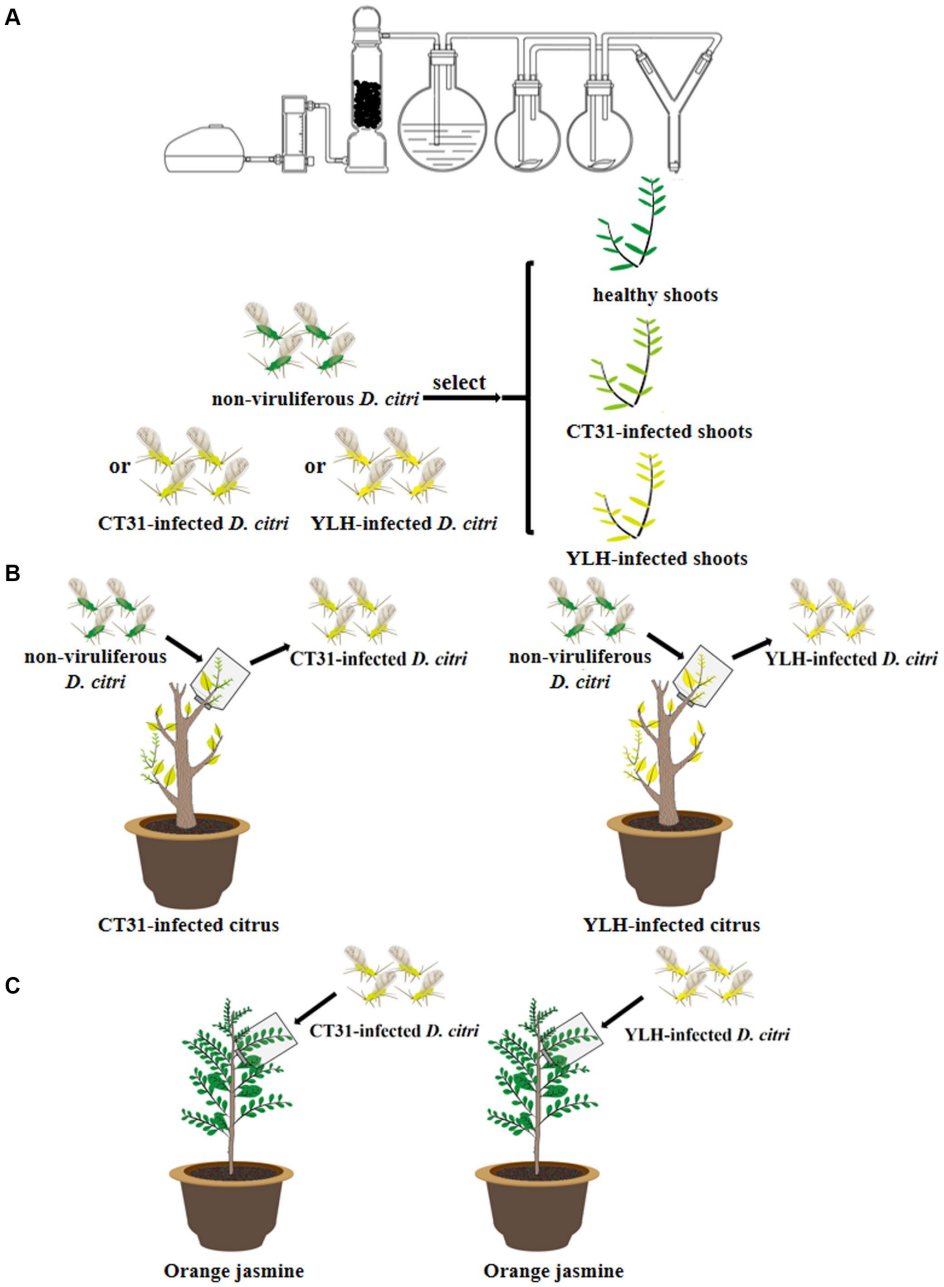
Diagram of the main experiments in this study. **(A)** Plant selection preference assay by *Diaphorina citri*. **(B)** Acquisition of different CTV isolates by *D. citri*. **(C)** Persistence of different CTV isolates by *D. citri*.

A Y-shaped glass tube olfactometer was applied to record the responses of different *D. citri* to different plants following the method described by Signoretti et al. (2012) and Yi et al. (2021), with minor modifications. Young psyllid adults (1–7 days after emergence) were screened for the following experiments. Non-viruliferous *D. citri* adults, *D. citri* adult carrying CT31 (with infection rate of 90% and average Ct value of 26.89 ± 1.05) and *D. citri* adult carrying YLH (with infection rate of 90% and average Ct value of 27.09 ± 0.75) were randomly selected for matched-pair analysis. Separate analyses were undertaken for the following three comparisons (Figure 1A): (a) the preference of non-viruliferous *D. citri* for CT31-infected, YLH-infected and healthy shoots, (b) the preference of CT31-infected *D. citri* for CT31-infected, YLH-infected and healthy shoots and (c) the preference of YLH-infected *D. citri* for CT31-infected, YLH-infected and healthy shoots. Experiments were conducted at a room temperature of 25 ± 2°C at day time. Insects were tested after starving for 3 h. Specifically, *D. citri* was released into the lower arm (served as an entry) of the Y-shaped tube one by one, and the moving behavior was recorded for 5 min. The position of two odor sources was exchanged regularly for each assay to avoid position bias. The odor source was considered to be selected if psyllid crawled through 1/3 of the source arm within 5 min and stayed there for more than 30 s. Selection results of a total of 100 adult individuals in each group were recorded. Each assay was replicated 5 times, with 10 females and 10 males being used in each replicate. Insects were not reused in the test. After each trial, the olfactometer was disinfected with 90% ethanol (v/v) and rinsed with deionized water.

### Acquisition and persistence of different CTV isolates by *Diaphorina citri*

A total of 600 non-viruliferous young adult *D. citri*, which had been starved for 3 h, were placed on CT31-infected or YLH-infected seedlings (Figure 1B). After feeding for 1 min, 3 min, 0.5 h, 2 h, 0.5 d, 1 d, 2 d, 3 d, 7 d, 14 d, and 28 d acquisition access period (AAP), 20 *D. citri* were collected at each time point. The same number of psyllids on three healthy plants at these AAPs were selected as controls. RNA of collected insects was extracted for qPCR detection of CTV. Each assay was replicated 3 times.

A total of 300 non-viruliferous newly emerged adult *D. citri* were divided into two groups and fed on CT31-infected or YLH-infected seedlings for 3 days. The CTV acquisition rate of the two groups reached more than 85% after 3 d AAP by qRT-PCR detection. The two groups of viruliferous psyllids were subsequently transferred onto healthy orange jasmine plants for persistence rearing (Figure 1C). After 3, 6, 12, and 24 days of feeding (recorded as the day after AAP, d AAAP), 30 psyllids were collected each for RNA extraction and qPCR detection. Each assay was replicated 3 times.

### Detection of endosymbionts in *Diaphorina citri*

About 150 non-viruliferous young *D. citri* adult individuals were fed on CT31-infected, YLH-infected or healthy citrus seedlings, respectively. 20 adult psyllids were collected from each group of plants at 0.5 d, 1 d, 3 d, 5 d, 7 d, 14 d and 28 d AAP. For the CTV persistence assay, groups of the CT31-exposed or YLH-exposed *D. citri* adults (with more than 85% individuals infected) were transferred onto CTV-free orange jasmine trees for feeding. Another five sampling events (1, 3, 6, 12, and 24 d AAAP) with 30 psyllids were performed during the feeding time. Total DNA of psyllid individuals was extracted. Specific primers (Table 1) for the “*Ca.* Carsonella ruddii,” “*Ca.* Profftella armatura” and *Wolbachia* were used for quantitative PCR analysis of the endosymbionts. Each assay was replicated 3 times.

### Statistical analyses

The results were analyzed statistically using Microsoft Excel 2019 and IBM SPSS Statistics (version 26.0, IBM Corporation, Armonk, NY, United States). Independent t-test was used to evaluate the differences in terms of host selection in two different *D. citri* populations (*p* < 0.05). Pathogen or endosymbiont titers of different AAP or dAAAP were subjected to statistical analysis by one-way analysis of variance (ANOVA) followed by Duncan’s new multiple range test. Different letters in the figures indicate significant differences at *p* < 0.05 level.

## Results

### Effect of CTV on host preference of *Diaphorina citri*

**Figure 2:**
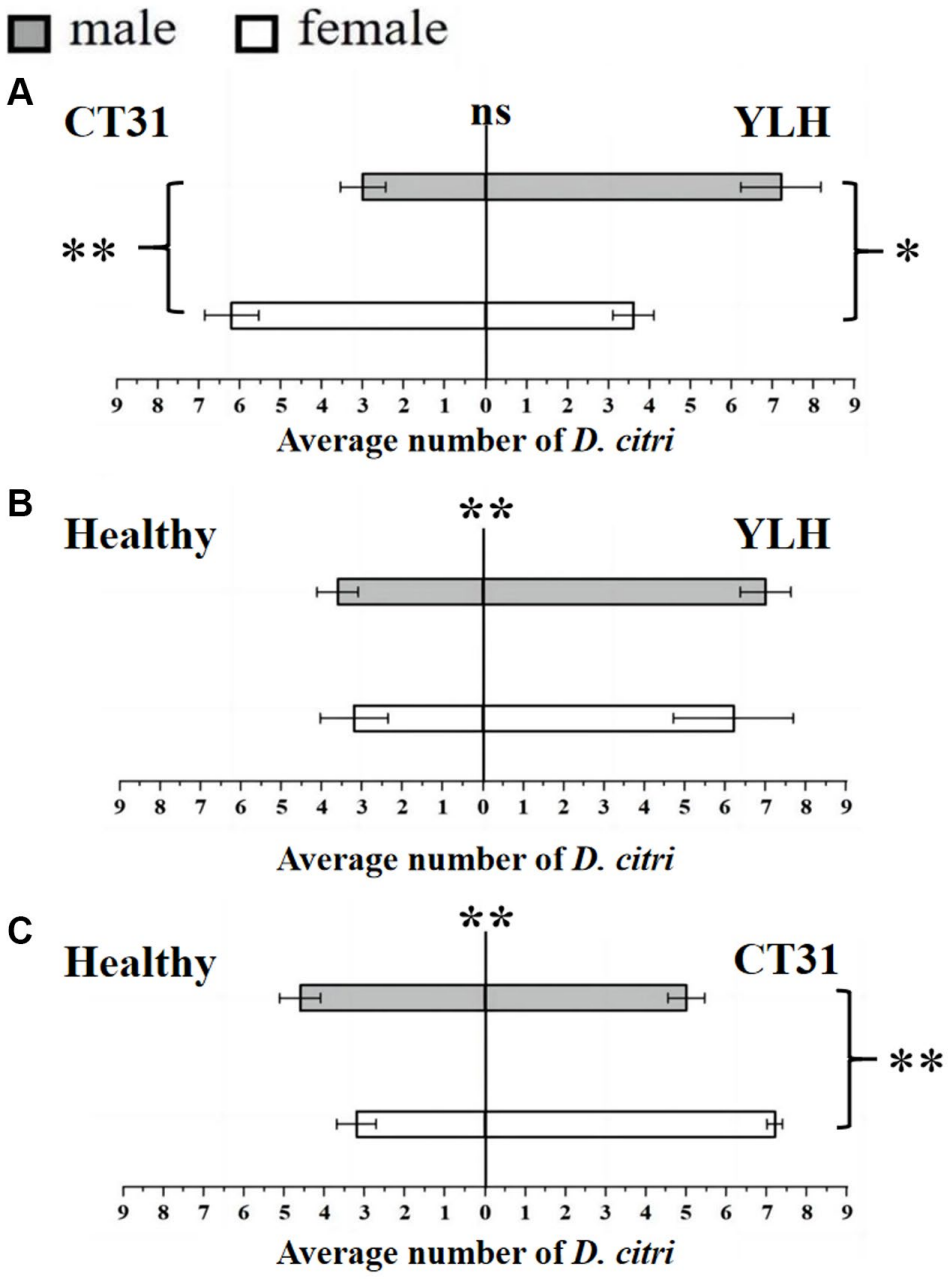
Preference of non-viruliferous *D. citri* for CT31-infected, YLH-infected and healthy citrus shoots. **(A)** The non-viruliferous *D. citri* selected CT31-infected or YLH-infected shoots. **(B)** The non-viruliferous *D. citri* selected healthy or YLH-infected shoots. **(C)** The non-viruliferous *D. citri* selected healthy or CT31-infected shoots. The numbers under the abscissa axis represent the number of psyllids. Bars represent means with standard errors (SE). The asterisk indicates a significant preference within an independent t-test: ns, not significant; *, *p* < 0.05; **, *p* < 0.01. The significant preference in the middle of each figure indicates the difference in the selection of different citrus shoots by *D. citri*.

Results for the Y-shaped olfactometer bioassays on determining the selection behavior of non-viruliferous *D. citri,* against CT31-infected or YLH-infected shoots, showed that there was no significant difference in response between the two (*p* = 0.088). The average number of *D. citri* attracted by CT31-infected citrus shoots was 9.2 ± 0.5, while that attracted by YLH-infected shoots was 10.8 ± 0.58. For CT31-infected shoots, females were significantly more selective than males (*p* = 0.006), with 6.2 ± 0.66 and 3.0 ± 0.55, respectively. However, for *D. citri* selected YLH-infected shoots, the number of male (7.2 ± 0.97) was significantly more than that of female (3.6 ± 0.51) (*p* = 0.011) (Figure 2A).

In summary, there was no difference in the selection of different CTV strains among the non-viruliferous adult *D. citri.* Among the psyllids that selected CT31-infected shoots, females were significantly more selective than males, while among those selected YLH-infected shoots, males were significantly more than females.

The selection by non-viruliferous *D. citri* between YLH-infected shoots and healthy shoots was compared. The results demonstrated that YLH-infected shoots were more likely to attract psyllids (*p* = 0.001), but there was no gender difference in selection for both ends of the tube (*p* = 0.545 and *p* = 0.408) (Figure 2B). Similarly, compared to the healthy plants, odors from CT31-infected shoots were more attractive to non-viruliferous psyllids (*p* = 0.001), especially the females (*p* = 0.002) (Figure 2C).

In conclusion, non-viruliferous *D. citri* preferred to select the shoots with CTV, but different strains did not affect their selection. Among the psyllids that selected CT31-infected shoots, females were significantly more common than males. There was no gender difference among the psyllids that chose YLH-infected shoots.

**Figure 3:**
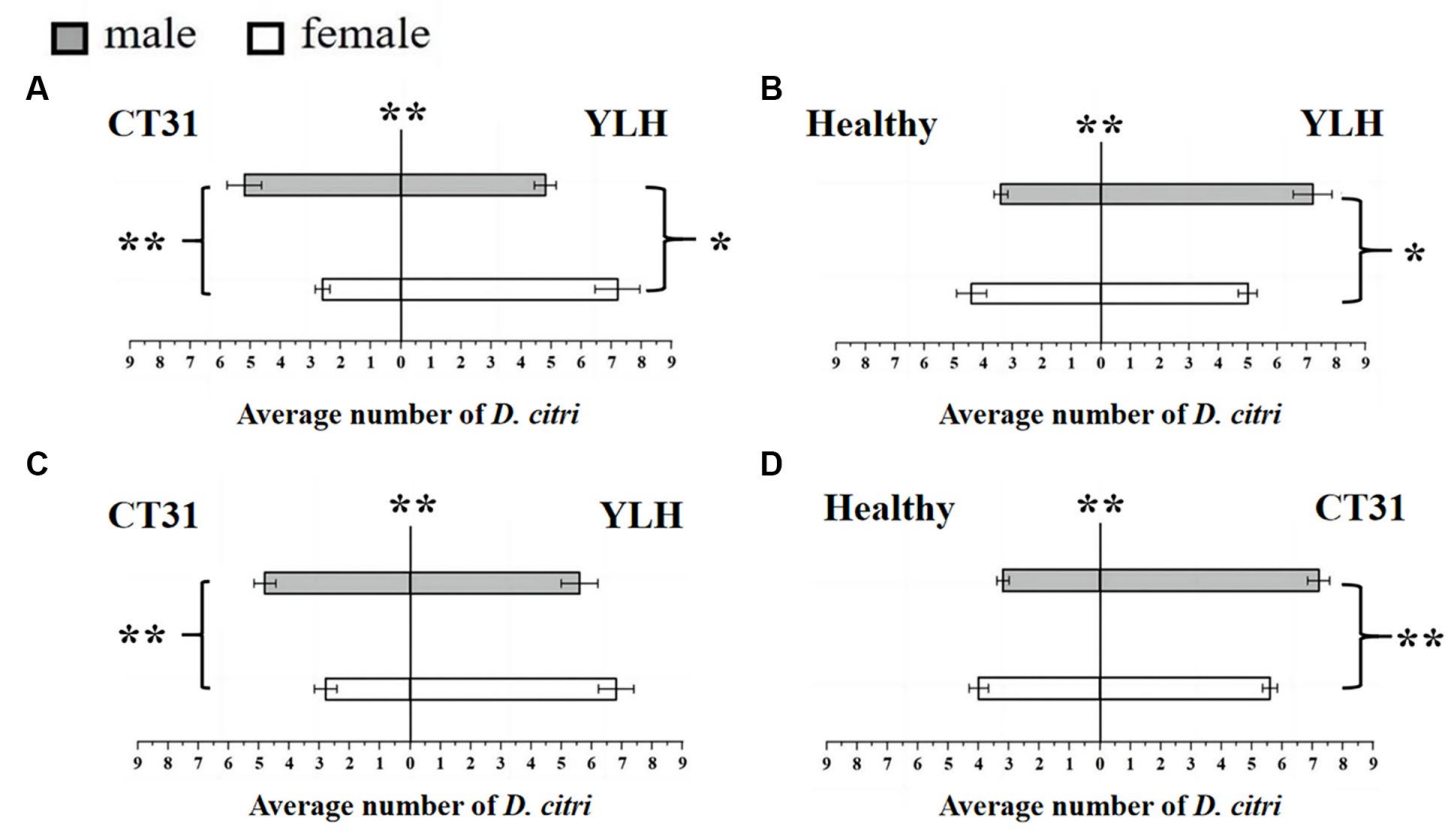
Preference of CT31-infected or YLH-infected *D. citri* for CT31-infected, YLH-infected and healthy citrus shoots. **(A)** The CT31-infected *D. citri* selected CT31-infected or YLH-infected shoots. **(B)** The CT31-infected *D. citri* selected healthy or YLH-infected shoots. **(C)** The YLH-infected *D. citri* selected CT31-infected or YLH-infected shoots. **(D)** The YLH-infected *D. citri* selected healthy or CT31-infected shoots. Bars represent means with standard errors (SE). The asterisk indicates a significant preference within an independent t-test: *, *p* < 0.05; **, *p* < 0.01. The significant preference in the middle of each figure indicates the difference in the selection of different citrus shoots by *D. citri*.

Bioassays were also conducted using a Y-shaped olfactometer to determine the selection behavior of CT31-infected *D. citri* against CT31-infected or YLH-infected shoots. The results showed that the average number of *D. citri* attracted by odors from YLH-infected shoots was 12.2 ± 0.58, which was significantly higher than those attracted by the odors of CT31-infected shoots (7.8 ± 0.58) (*p* = 0.001). For those selecting YLH-infected shoots, females were considerably more common than males (*p* = 0.014) (Figure 3A). However, significantly more males preferred odors of CT31-infected plants than females (*p* = 0.003). Similarly, the shoots infected with YLH were more likely to attract CT31-infected *D. citri* than healthy shoots. In the population that selected YLH-infected shoots, males were significantly more common than females (*p* = 0.017) **(**Figure 3B**)**.

The selection behavior of *D. citri* carrying YLH on CT31-infected or YLH-infected shoots was also determined. The results showed that YLH-infected *D. citri* tended to choose the side with YLH-infected shoots, but there was no gender difference in the *D. citri* (*p* = 0.189). However, among the *D. citri* that selected CT31-infected shoots, males were significantly more present than females (*p* = 0.005) **(**Figure 3C**)**. Meanwhile, in the comparison between CT31-infected and healthy shoots, the former were more likely to attract YLH-infected *D. citri* individuals. Among the selecting insects, males were significantly more common than females (*p* = 0.007) (Figure 3D).

In conclusion, the *D. citri* that carried CTV, regardless of CT31 or YLH, showed a significant preference for infected plants compared to non-viruliferous plants, especially the males. Compared to CT31-infected plants, YLH-infected seedlings were more attractive to both *D. citri* individuals carrying CT31 and those carrying YLH.

### Acquisition efficiency of different CTV isolates by groups of *Diaphorina citri*

**Figure 4:**
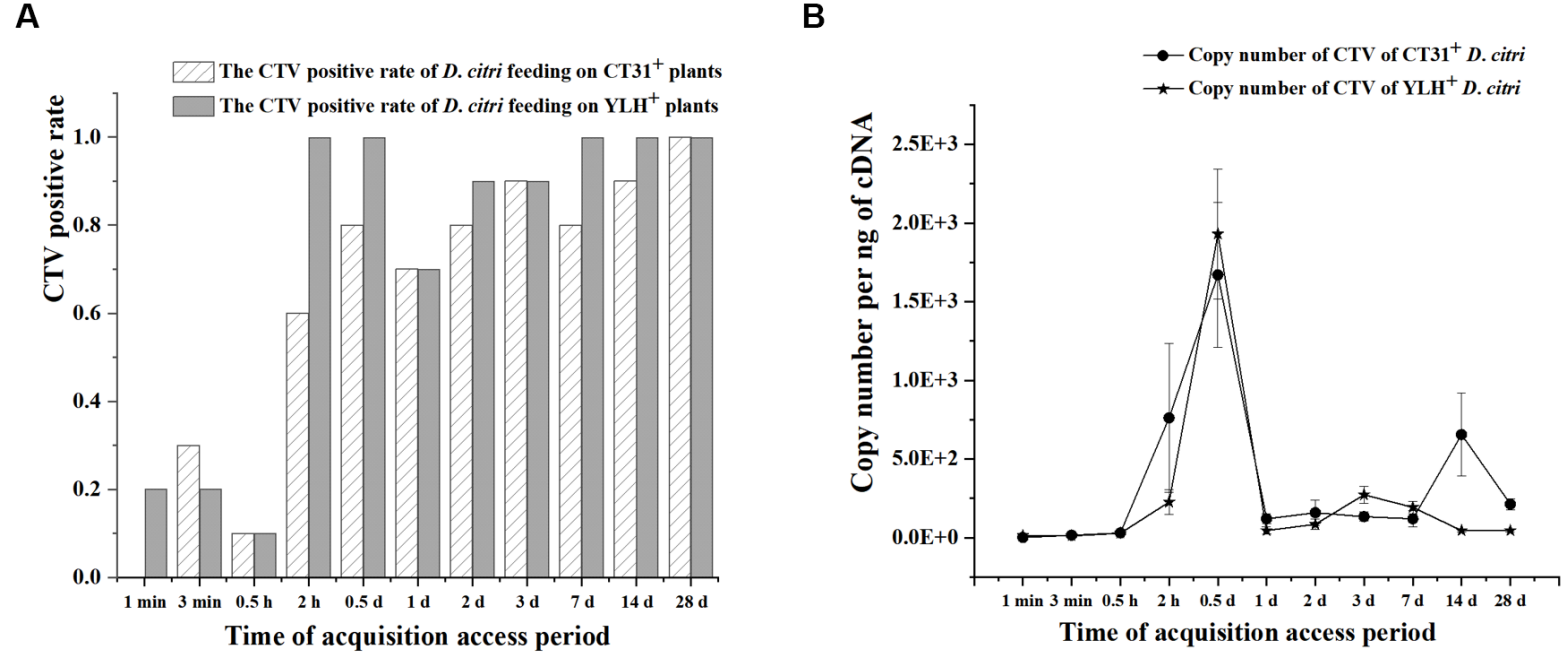
The acquisition efficiency of CTV by *D. citri* during an acquisition assessment period of 28 d on CT31- or YLH-infected “Shatangju” mandarin plants. **(A)** The CTV acquisition rate of CT31 or YLH in *D. citri*. **(B)** The copy number of CT31 or YLH obtained in *D. citri*. Bars represent means with standard errors (SE) of n = 20*3. The asterisk indicates a significant differences within an independent t-test: *, *p* < 0.05; **, *p* < 0.01.

The infection rate increased with feeding time in non-viruliferous *D. citri* populations feeding on CT31-infected seedlings or YLH-infected seedlings **(**Figure 4A**)**. Within 12 h of feeding on CT31-infected or YLH-infected seedlings, the abundance of CTV increased continuously. The amount of CTV was stable during 1 d AAP and 28 d AAP, and the overall trend was consistent between the two strains **(**Figure 4B**)**. The Ct value in CTV detection was the lowest at 0.5 d AAP. At 1 min, the CTV Ct value of *D. citri* populations feeding on YLH-infected seedlings was significantly lower than those on CT31-infected seedlings (*p* = 0.001). At 1 d AAP, 14 d AAP and 28 d AAP, the CTV Ct values of *D. citri* feeding on YLH-infected seedlings were significantly higher than those feeding on CT31-infected seedlings (*p* < 0.05).

After feeding on CT31-infected seedlings, the copy number of CTV in *D. citri* populations of different feeding time was significantly different (*p* = 0.001) (Figure 4B**)**. The copy number of CTV in *D. citri* increased sharply during 0.5 h AAP and 0.5 d AAP, but decreased afterwards until 1 d AAP, and then fluctuated steadily. A similar pattern was observed for *D. citri* feeding on YLH-infected seedlings. In addition, the copy number of CTV in *D. citri* feeding on YLH-infected seedlings increased more significantly during 0.5 h and 0.5 d AAP, with a higher copy number of CTV than that of *D. citri* feeding on CT31-infected seedlings at 0.5 d AAP.

Overall, the infection rate of *D. citri* increased with feeding time (1 min AAP to 28 d AAP) for both groups of insects on plants infected with different CTV isolates. With the extension of feeding time, the abundance of CTV in *D. citri* reached a significant peak at 0.5 d AAP.

### Persistence of different CTV isolates in *Diaphorina citri*

**Figure 5:**
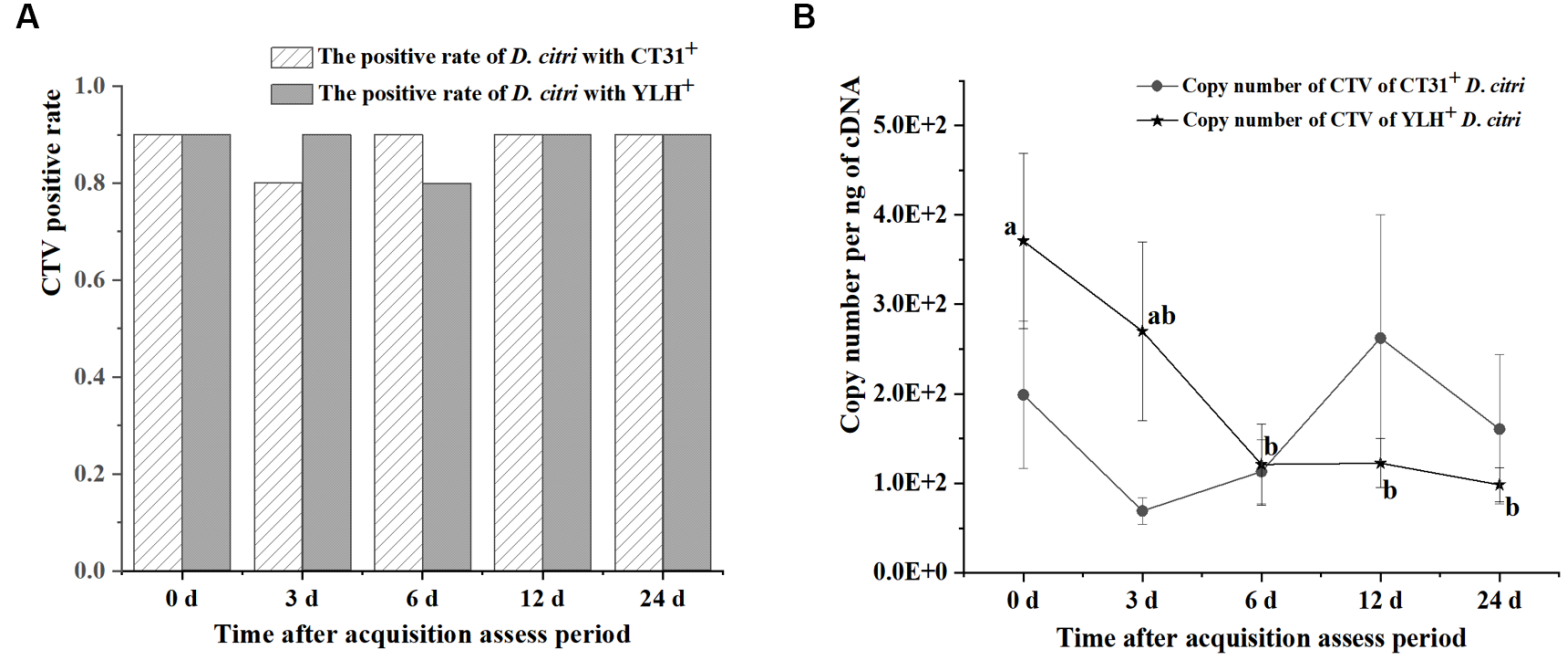
The persistence of two CTV isolates (CT31 and YLH) by *D. citri* during a period of 24 d on orange jasmine (*Murraya paniculata* L.) plants. **(A)** The infection rate of *D. citri* with CT31 or YLH. **(B)** The copy number of CT31 or YLH in *D. citri*. Bars represent means with standard errors (SE) of n = 30*3. Different letters indicated significant difference in the copy number of CTV in YLH-infected *D. citri* at different time periods at the *p* < 0.05. There was no significant difference in the copy number of CT31-infected *D. citri* at different time periods.

After rearing on CT31-infected and YLH-infected seedlings for 3 d AAP, psyllids were transferred to healthy orange jasmine plants for further experimentation. The results showed that the CTV-infected rates of the *D. citri* populations with either CTV isolate were above 80% during 3 d AAAP to 24 d AAAP on orange jasmine plants (Figure 5A). Although the Ct values ranged from 24.46 to 31.77 for the *D. citri* individuals in a group carrying CT31 and from 25.21 to 31.18 for the group carrying YLH (from 3 d AAAP to 12 d AAAP), but there was no significant difference in Ct values within the same group. There was no significant difference in Ct values of CTV between CT31-infected and YLH-infected *D. citri* populations when feeding on orange jasmine plants for the same duration (Figure 5A). The copy number of CTV in CT31-infected *D. citri* decreased from 0 d AAAP to 3 d AAAP, increased from 3 d AAAP to 12 d AAAP and then decreased again from 12 d AAAP to 24 d AAAP, but there was no significant difference (*p* = 0.552) among them (Figure 5B). Comparatively, the copy number of CTV in YLH-infected *D. citri* showed a decreasing trend from 3 d AAAP to 24 d AAAP, without significant difference (*p* = 0.113). However, the copy numbers of YLH at the last three time points were significantly lower than that immediately after AAP (0 d AAAP). Similarly, there was no significant difference in the copy number of CTV in *D. citri* carrying different CTV isolates after rearing on the orange jasmine plants for the same time.

Taken together, the CTV infection rates of *D. citri* did not significantly change when the infected *D. citri* populations were transferred to non-viruliferous orange jasmine plants, which is a non-host for CTV. There were no significant differences in the Ct values within a population at different times or between populations at the same time. A decrease only found in the copy numbers of YLH from 6 d AAAP to 24 d AAAP comparing to that at 0 d AAAP.

### Infection titers of endosymbionts in different CTV-exposed and unexposed adult *Diaphorina citri*

**Figure 6:**
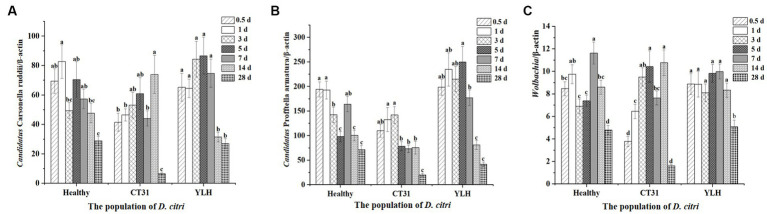
Relative titers of three endosymbionts in *D. citri* after CTV acquisition during a 28-day acquisition access period on “Shatangju” mandarin plants. **(A)** Relative titers of *Candidatus* Carsonella ruddii. **(B)** Relative titers of *Candidatus* Profftella armatura. **(C)** Relative titers of *Wolbachia*. Bars represent means with standard errors (SE) of n = 20*3. Different letters indicated that the relative persistence of endosymbionts of *D. citri* at different times had significant differences at *p* < 0.05 using by Duncan’s new multiple range test.

After rearing the *D. citri* populations separately on *Citrus reticulata* Blanco seedlings infected with CT31 or YLH, or on healthy plants, the insects collected at different times of AAP were used to quantify three endosymbionts. It was found that there was a significant difference in the relative titers of “*Ca.* Carsonella ruddii” in *D. citri* at different times of AAP (*p* < 0.001) (Figure 6A). Although no significant CTV titer variation was observed during 1–28 d AAP, the density of “*Ca.* Carsonella ruddii” was significantly lower at the late stage (28 d AAP) as compared to those from 0.5 d to 14 d AAP (*p* < 0.001). Comparatively, relative titers of “*Ca.* Carsonella ruddii” in *D. citri* feeding on YLH-infected seedlings were higher than those on CT31-infected seedlings.

The relative titers of “*Ca.* Profftella armatura” in *D. citri* during AAP were detected (Figure 6B). Similarly, the “*Ca.* Profftella armatura” titers were significantly reduced at the later stages of AAP, especially at 14 d AAP and 28 d AAP (*p* < 0.001). The infection titers of “*Ca.* Profftella armatura” for the YLH-exposed *D. citri* population were higher, while those in the CT31-exposed *D. citri* population were relatively lower than the controlled unexposed *D. citri* group.

The relative titers of *Wolbachia* in the three *D. citri* populations varied across different times of AAP (*p* < 0.001). *Wolbachia* titers were significantly lower at 28 d AAP, for either the CTV-exposed or unexposed *D. citri* populations, than those collected from 0.5 d AAP to 14 d AAP (Figure 6C). Additionally, YLH-exposed insects showed significantly higher titers of *Wolbachia* in bacterium than most of the same time points of AAP in unexposed insects.

In conclusion, the relative titers of a given endosymbiont varied in *D. citri* populations of CTV-exposed and unexposed during the AAP. Similar profiles were assessed for “*Ca.* Carsonella ruddii,” “*Ca.* Profftella armatura,” and *Wolbachia*. An initially increasing trend followed by a decreasing trend was consistent for the three endosymbionts across the AAP.

**Figure 7:**
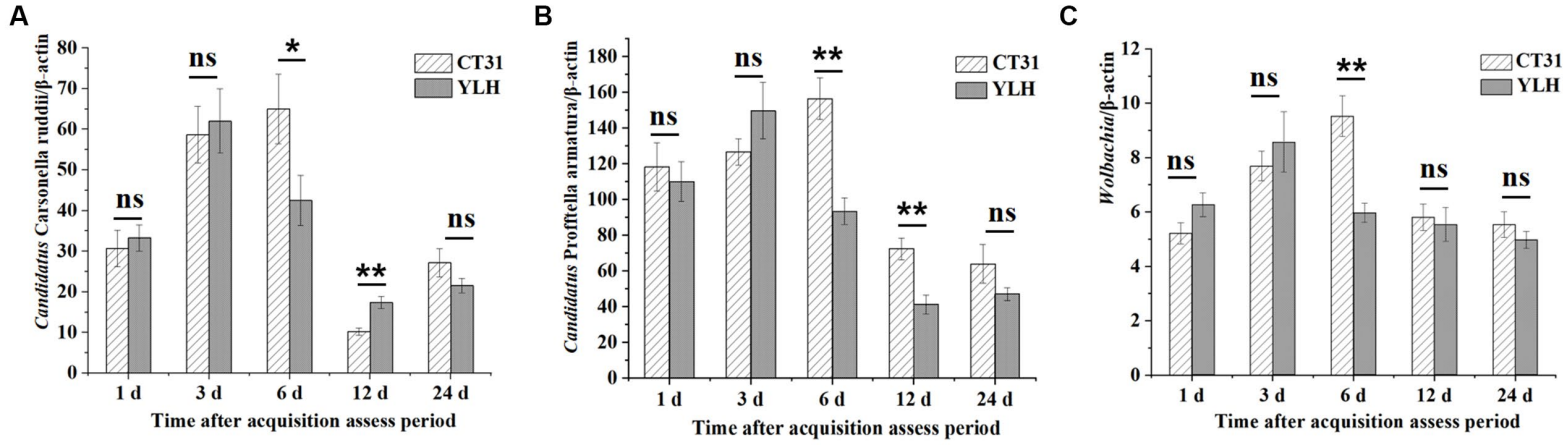
Relative titers of three endosymbionts in CT31 or YLH-exposed adult *D. citri* during CTV persistence period on orange jasmine (*Murraya paniculata* L.) plants. **(A)** Relative titers of *Candidatus* Carsonella ruddii. **(B)** Relative titers of *Candidatus* Profftella armatura. **(C)** Relative titers of *Wolbachia*. Bars represent means with standard errors (SE) of n = 30*3. The asterisk indicates a significant differences within an independent t-test: ns, not significant; *, *p* < 0.05; **, *p* < 0.01.

During the CTV persistence, the relative titers of “*Ca.* Carsonella ruddii” and *Wolbachia* in *D. citri* infected with CT31 or YLH first increased and then decreased (Figures 7A,C). Differently, a decrease trend for the “*Ca.* Profftella armatura” titers during the persistence were observed. After AAP, the relative titers of “*Ca.* Carsonella ruddii” in CT31-infected psyllids were higher at 3 d AAAP and 6 d AAAP but lower at 12 d AAAP and 24 d AAAP (Figure 7A). The same pattern was observed when the YLH-infected psyllids fed on non-viruliferous non-host orange jasmine plants. When comparing the “*Ca.* Carsonella ruddii” titers between CT31-exposed and YLH-exposed *D. citri* adults, significantly greater titers of “*Ca.* Carsonella ruddii” were detected in the former at 6 d AAAP. However, the results were the opposite at 12 d AAAP (Figure 7A).

Different from the measurement for “*Ca.* Carsonella ruddii,” the titers of the other two endosymbionts were remarkably lower in the later stages of 12 and 24 d AAAP (Figures 7B,C). At 6 d AAAP and 12 d AAAP, the relative titers of “*Ca.* Profftella armatura” in CT31-infected *D. citri* were significantly higher than those of YLH-infected *D. citri*. The relative titer of *Wolbachia* in CT31-infected *D. citri* was the highest at 6 d AAAP, and the relative titer of *Wolbachia* in YLH-infected *D. citri* was the largest at 3 d AAAP. At 6 d AAAP, the relative titer of *Wolbachia* in CT31-infected *D. citri* was significantly higher than in YLH-infected *D. citri* (*p* < 0.001).

In conclusion, the relative titers of the three endosymbionts in *D. citri* varied significantly with different durations of CTV persistence, although the CTV titers showed no significant differences. The relative titers of the three endosymbionts decreased in the later process of CTV persistence. Variation patterns were similar for “*Ca.* Carsonella ruddii” and *Wolbachia*, which were suggest to be negatively correlated to the CT31 titers. An earlier increment of the endosymbiont titers was observed in the YLH-exposed *D. citri* populations.

## Discussion

Studies have found that host plants infected with viruses are more attractive to insect vectors than healthy host plants (Ng and Falk, 2006; Mauck et al., 2010, 2012; McMenemy et al., 2012). In this study, the non-viruliferous *D. citri* individuals preferred CT31- or YLH-infected citrus compared to the healthy plants. This explains the extensive distribution of trees infected with CTV and the high rates of CTV-infected psyllids in the field (Britt et al., 2020). We previously found that the acquisition and transmission of CLas by *D. citri* was facilitated by CTV (Chen et al., 2023). Adding toghther, we are more certain that the high efficiency of CLas transmission in the field is related this virus. A previous study showed that plants infected with CMV attracted non-viruliferous aphids, while infected aphids rejected feeding on infected plants (Mauck et al., 2010). However, our results found that CTV-exposed *D. citri* preferred YLH-infected plants. In 2013, Webster et al. (2013) proposed that insects preferentially respond to volatile odors that they remember when the odor source is easily accessible, which may explain why the infected *D. citri* often return to the viruliferous plants. YLH is a mixture of different CTV isolates, which work as a plant vaccine to cross-protect citrus tristeza disease in the citrus industry. Stockton et al. (2016) demonstrated that male and female psyllids are dynamic animals that displayed differing discriminatory abilities. The way they perform in host plant preference, learn to recognize olfactory and visual host plant stimuli were sex specific. In this study, female *D. citri* tended to choose the milder CTV strain of CT31. It is hypothesized that the less affected plants support the egg laying and reproduction of the psyllid population. This hypothesis needs to be further tested.

By using an Electrical Penetration Graph (EPG) assay, Zhou et al. (2018) suggested that virus infection-triggered plant cues would influence feeding behavior responses of the vectors, which directly influenced the virus acquisition and transmission efficiency of the insects. Tomato plants infected by TYLCV had a lower level of resistance and an increased callose accumulation, which helped the survival and virus-acquiring of *Bemisia tabaci* on them (Su et al., 2015). Callose deposition was found promoted in *C.* × *sinensis* infected with the CTV severe isolate B6 (Fu et al., 2016) or infected with CLas (Fu et al., 2015). In this study, at some testing times during AAP, there were significant differences in the virus densities of the two CTV strains in *D. citri*. Previous studies have shown that different virus strains affected insect vectors differently associated with the host defense system, which is mediated by different viruses (Wang et al., 2020). Alternatively, it may be related to the different changes in metabolites of plants after infection by pathogens (Mauck et al., 2016, 2018; Eigenbrode et al., 2018). In this study, CT31- and YLH-mediated changes in the metabolite level of the plants were different, resulting in differences in the acquisition and persistence of the two isolates by *D. citri*.

“*Ca.* Carsonella ruddii,” “*Ca.* Profftella armatura” and *Wolbachia* were detected in all samples of *D. citri* at different time of AAP or after AAP. As previously mentioned, these three endosymbionts were harbored by *D. citri.* As such, they influenced *D. citri* physiology and fitness (Chu et al., 2016). Consistent with this study, the relative titers of the three endosymbionts generally had positive correlation with each other. “*Ca.* Carsonella ruddii” has a high degree of co-speciation and dependence on host insects (Koga et al., 2012; Sloan and Moran, 2012; Balmand et al., 2013). Likewise, “*Ca.* Carsonella ruddii” was found in all 12 assessed psyllid species (Nakabachi et al., 2022). The probable negative co-relationship between “*Ca.* Carsonella ruddii” and CTV was observed in the CTV persistence access period. Chu et al. (2016) indicated that the densities of “*Ca.* Carsonella ruddii” were significantly lower in CLas-positive compared to CLas-negative *D. citri* population. Furthermore, Chen et al. (2023) reported that CTV promoted the acquisition and transmission of CLas by *D. citri*. Therefore, we believe that the inhibition of “*Ca.* Carsonella ruddii” reduces nutrition (Thao et al., 2000; Nakabachi et al., 2006) and proliferation of CTV in *D. citri*, which might facilitate the efficiency of *D. citri* to acquire CLas.

“*Ca.* Profftella armatura,” which promotes insect defenses, is extremely abundant in *D. citri* (Nakabachi et al., 2013). Previous studies have shown that the abundance of “*Ca.* Profftella armatura” and “*Ca.* Carsonella ruddii” were significantly reduced in *D. citri* feeding on CLas-infected plants (Hosseinzadeh et al., 2019). This study found that the relative titers of “*Ca.* Profftella armatura” in YLH-infected *D. citri* were higher than CT31-infected *D. citri* during AAP, however, higher abundance of it was found in the CT31-infected *D. citri* as compared to that in the YLH-infected insects during the persistence assess period. It is speculated that “*Ca.* Profftella armatura” promotes defensive effects in *D. citri* and somewhat inhibits the proliferation of the mixed CTV strain YLH. Most reports show that *Wolbachia* is associated with regulating reproduction (Werren et al., 2008; Correa and Ballard, 2012). Through experiments, it was found that the titers of *Wolbachia* decreased with the CT31 persistence, and it was speculated that its reproductive function was also affected. These results provide a better understanding of multi-trophic interactions regulating symbiont dynamics in the HLB pathosystem.

In recent years, our laboratory found that *D. citri* in the field can carry CTV and persist CTV for 15 d, and CTV-infected *D. citri* can transmit CTV to healthy plants after 15 d of feeding (Wu et al., 2021). This study suggests the CTV-positive incidence and the average CTV titers in the *D. citri* population that were transferred to orange jasmine plants were reduced after 5- and 15-d post AAP. However, this study proposed that both the incidence and the CTV titers were not significantly reduced with time once the insects were transferred onto orange jasmine plants after the AAP. Although more evidence should be acquired to determine whether *D. citri* does, in fact, transmit CTV, our data provide a preliminary indication that the virus would be kept in the body of psyllids for longer than 24 days. Collectively, in this study, we determined the preference of *D. citri* for CTV-infected plants, analyzed the relationship between three endosymbionts of *D. citri* and CTV, and compared the differences in the acquisition and persistence of the two strains of CTV of *D. citri*. Overall, this explains some of the reasons for the influence of CTV on the transmission of CLas by *D. citri*.

## Data availability statement

The raw data supporting the conclusions of this article will be made available by the authors, without undue reservation.

## Author contributions

MX designed the study, helped write the manuscript, and acquired the funding. XC and YL wrote the draft manuscript, finished most of the experiments, and conducted the data. JZ helped collected and extracted the psyllid samples for this study. PH helped the DNA and RNA extraction. ZZ helped designed the study and helped revise the manuscript. XD helped the funding acquisition. All authors contributed to the article and approved the submitted version.

## Funding

This research was funded by the Natural Science Foundation of Guangdong Province, grant number 2022A1515010889 and the open competition program of top ten critical priorities of Agricultural Science and Technology Innovation for the 14th Five-Year Plan of Guangdong Province (2022SDZG06).

## Conflict of interest

The authors declare that the research was conducted in the absence of any commercial or financial relationships that could be construed as a potential conflict of interest.

## Publisher’s note

All claims expressed in this article are solely those of the authors and do not necessarily represent those of their affiliated organizations, or those of the publisher, the editors and the reviewers. Any product that may be evaluated in this article, or claim that may be made by its manufacturer, is not guaranteed or endorsed by the publisher.
